# Ultrastructural plasticity in the plant-parasitic nematode, *Bursaphelenchus xylophilus*

**DOI:** 10.1038/s41598-020-68503-3

**Published:** 2020-07-14

**Authors:** Taisuke Ekino, Haru Kirino, Natsumi Kanzaki, Ryoji Shinya

**Affiliations:** 1 School of Agriculture, Meiji University, Kawasaki, Kanagawa 214-8571 Japan; 2 Kansai Research Center, Forestry and Forest Products Research Institute (FFPRI), Kyoto, Kyoto 612-0855 Japan; 3 JST PRESTO, Meiji University, Kawasaki, Kanagawa 214-8571 Japan

**Keywords:** Evolution, Zoology, Parasite biology

## Abstract

Phenotypic plasticity is one of the most important strategies used by organisms with low mobility to survive in fluctuating environments. Phenotypic plasticity plays a vital role in nematodes because they have small bodies and lack wings or legs and thus, cannot move far by themselves. *Bursaphelenchus xylophilus*, the pathogenic nematode species that causes pine wilt disease, experiences fluctuating conditions throughout their life history; i.e., in both the phytophagous and mycetophagous phases. However, whether the functional morphology changes between the life phases of *B. xylophilus* remains unknown. Our study revealed differences in the ultrastructure of *B. xylophilus* between the two phases. Well-developed lateral alae and atrophied intestinal microvilli were observed in the phytophagous phase compared with the mycetophagous phase. The ultrastructure in the phytophagous phase was morphologically similar to that at the dauer stage, which enables the larvae to survive in harsh environments. It suggests that the living tree represents a harsh environment for *B. xylophilus*, and ultrastructural phenotypic plasticity is a key strategy for *B. xylophilus* to survive in a living tree. In addition, ultrastructural observations of obligate plant-parasitic species closely related to *B. xylophilus* revealed that *B. xylophilus* may be in the process of adapting to feed on plant cells.

## Introduction

Many environments are heterogeneous and change continuously. Most highly mobile organisms are able to move around to find optimal or better habitats. For example, winged insects or birds can fly away from environments that become unfavorable on a seasonal basis. Mobile animals can migrate into a new environment for feeding and/or reproduction. On the other hand, low mobility or sessile organisms cannot escape from their habitat when it becomes unfavorable and often alter their phenotype to adapt to their changing habitat. This ability of a single individual to develop more than one phenotype is termed “phenotypic plasticity.” Phenotypic plasticity plays a key role in the survival and propagation of certain organisms^1,2^ in nature where large environmental fluctuations occur and could be one driver of evolution through the initiation of adaptive divergence, i.e., “plasticity-first” evolution^3^.

Phenotypic plasticity is well-studied in plants. Plants are sessile and cannot move even if the environment becomes unfavorable, making plasticity very important for their survival^4^; plants can recognize changes in their environment and alter their forms to adapt without moving. As an example, plant leaves are particularly plastic and exhibit great diversity in shape, size, and color in nature. Environmental factors, such as temperature, light quality and intensity, and humidity, all affect leaf morphology^5–7^. Phenotypic plasticity has also been reported in many animals. Many clones of *Daphnia pulex* develop thorns as antipredator devices in the presence of chemical signals from predators such as the insect *Chaoborus americanus*, and clones of *D. pulex* have been shown to develop thorns, known as neckteeth, until the end of the fourth juvenile instar, when they were exposed to chemical cues emitted from their predator, the *Chaoborus flavicans* larva^8–10^.

Nematoda is one of the most abundant and diverse groups of organisms on earth^11,12^. Nematodes have no wings or legs, and their body size is relatively small, resulting in a low dispersal capacity. Phenotypic plasticity is essential in nematodes because of their reduced mobility. The phenotypic plasticity of nematodes has been reported previously; for example, *Caenorhabditis elegans* enters a stress-resistant dauer stage in response to harsh environmental conditions^13^. Under optimum growth conditions, second-stage larvae (L2) develop into third-stage larvae, and then into adults. Under unfavorable growth conditions, L2 become dauer larvae, which enable survival and dispersal, and in this stage they are resistant to environmental stresses^14^.

The stomatal phenotypic plasticity of Diplogastridae nematodes has been well studied. *Pristionchus pacificus* has two feeding structures: a “wide-mouthed” eurystomatous morph with two large teeth and a “narrow-mouthed” stenostomatous morph with one small tooth. Eurystomatous animals can feed on other nematode species^15^, whereas stenostomatous animals have difficulty feeding on other nematodes, but are specialized as bacteria feeders when food bacteria are sufficient^16^.

Recently, stomatal phenotypic plasticity has been reported in another nematode clade, the genus *Bursaphelenchus*^17^. Most *Bursaphelenchus* feed on fungi, but a few species parasitize plants. *Bursaphelenchus* spp. commonly have a syringe-like feeding structure referred to as a “stylet” to pierce fungal and plant cells for nutrient uptake. However, *Bursaphelenchus sinensis*, which inhabits in dead pine trees, has a predatory form that has a stylet with a wider lumen than the mycetophagous form, and feeds on other nematodes.

The plant-parasitic nematode, *Bursaphelenchus xylophilus,* the causal pathogen of pine wilt disease, is thought to inhabit more fluctuating environments than *B. sinensis*. *B. xylophilus* mostly experiences two different environmental conditions, living pine trees and dead, moldy pine trees, whereas *B. sinensis* experiences only dead pine trees. Although no stomatal plasticity was observed in *B. xylophilus*^17^, *B. xylophilus* likely has environmentally sensitive traits. *B. xylophilus* uses epithelial cells and resin canal parenchyma cells as food sources in a living pine tree^18^. In this study, we refer to the phase in a living pine tree as the “phytophagous phase.” In many cases, infection by *B. xylophilus* causes pine wilt disease and results in the death of the infected tree. Various bacteria and fungi grow on the dead pine tree, and *B. xylophilus* feeds on the fungi and propagates rapidly^19^. Herein, we refer to this phase as the “mycetophagous phase.” In the fluctuating environment, *B. xylophilus* adapts its phenotype to survive. Thus, we compared the phenotypes between the phytophagous and mycetophagous phases, as this was the best method to understand the survival strategy of *B. xylophilus* in relation to its phenotypic plasticity. Tsai et al.^20^ showed that the expression of the collagen gene family differed significantly between these two phases, which indicated that the nematode exhibited morphological phenotypic plasticity. Although collagens are essential in structural formation and modification in nematodes^21^, compound microscopy revealed that the only morphological difference between these two phases was in the tail shape of adult females.

In this study, we used transmission electron microscopy (TEM) to investigate differences in the ultrastructure of the cuticle and intestines of *B. xylophilus* as these structures consist mainly of collagen^22,23^. Differences were examined between the phytophagous and mycetophagous phases, and the functional significance of any structural change is discussed. Furthermore, to understand the evolutionary adaptation of *B. xylophilus* to its host plant, we examined the internal ultrastructure and intestines of an obligate plant-parasitic species (phytophagous phase), *Schistonchus* sp., which is closely related to *B. xylophilus*, belongs to the family Aphelenchoididae, and evolved from soil-inhabiting mycetophagous species^24^. Findings were compared with those observed from the phytophagous phase of *B. xylophilus*.

## Results

Nematodes of *B. xylophilus* in the phytophagous phase were recovered from inoculated pine trees at 2, 3, and 4 weeks after inoculation. Throughout the sampling time, initial visible symptoms of disease onset were noted. The tips of the pine needles turned yellow, but the other needle parts remained green. No differences were observed in the ultrastructure of *B. xylophilus* between the three different time points (2, 3, and 4 weeks). Therefore, all the morphological information recorded during the phytophagous phase described hereafter was obtained from *B. xylophilus* recovered from inoculated pine trees at 2 weeks post-inoculation.

### Observations of ultrastructure using TEM

We observed the cuticle, lateral alae, and intestinal ultrastructure of *B. xylophilus* grown on live pines, dead pines inoculated with fungus, and on fungal cultures.

### Cuticle

**Figure 1:**
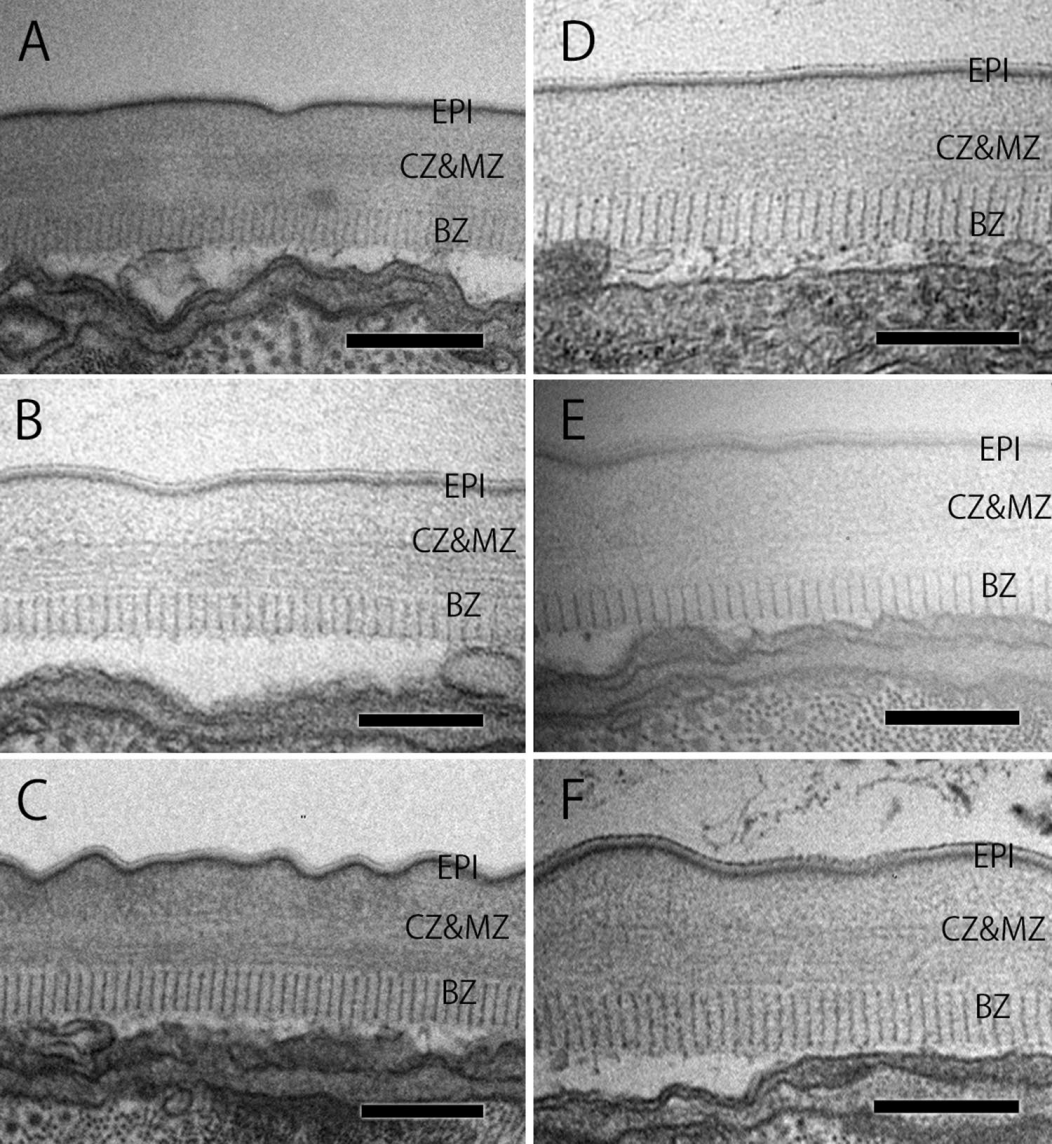
Cuticular structure in *Bursaphelenchus xylophilus* adults. (**A**) Female in the mycetophagous phase cultured on agar; (**B**) female in the mycetophagous phase cultured on pine stem; (**C**) female in the phytophagous phase; (**D**) male in the mycetophagous phase cultured on agar; (**E**) male in the mycetophagous phase cultured on pine stem; (**F**) male in the phytophagous phase. *EPI* epicuticle, *CZ & MZ* cortical zone and median zone, *BZ* basal zone. Scale bar = 200 nm.

Tsai et al. showed that the expression of the collagen gene family differed significantly between the mycetophagous phase and phytophagous phases^20^, which suggests that the cuticle, a structure consisting mainly of collagen, changes between the two phases. However, we observed no qualitative differences in the cuticular structure of *B. xylophilus* between sexes and phases (Fig. 1). The structure of the cuticle was morphologically the same as that previously reported^25^. It consisted of three parts: an epicuticle (EPI), cortical and median zones (CZ and MZ), and a basal zone (BZ). The EPI consisted of three layers: an electron-dense outermost layer (surface coat) and two inner layers. However, the triple layer was sometimes indistinct and appeared as a single or double layer. The CZ and MZ were electron-lucent and not distinguishable from each other, forming an amorphous zone. In comparison, the BZ was distinguishable from the MZ due to its radial striation. The thickness of the cuticle was significantly different between the two different phases, i.e., the phytophagous and mycetophagous phases cultured on agar plates for both females and males (P < 0.05).

### Lateral alae

**Figure 2:**
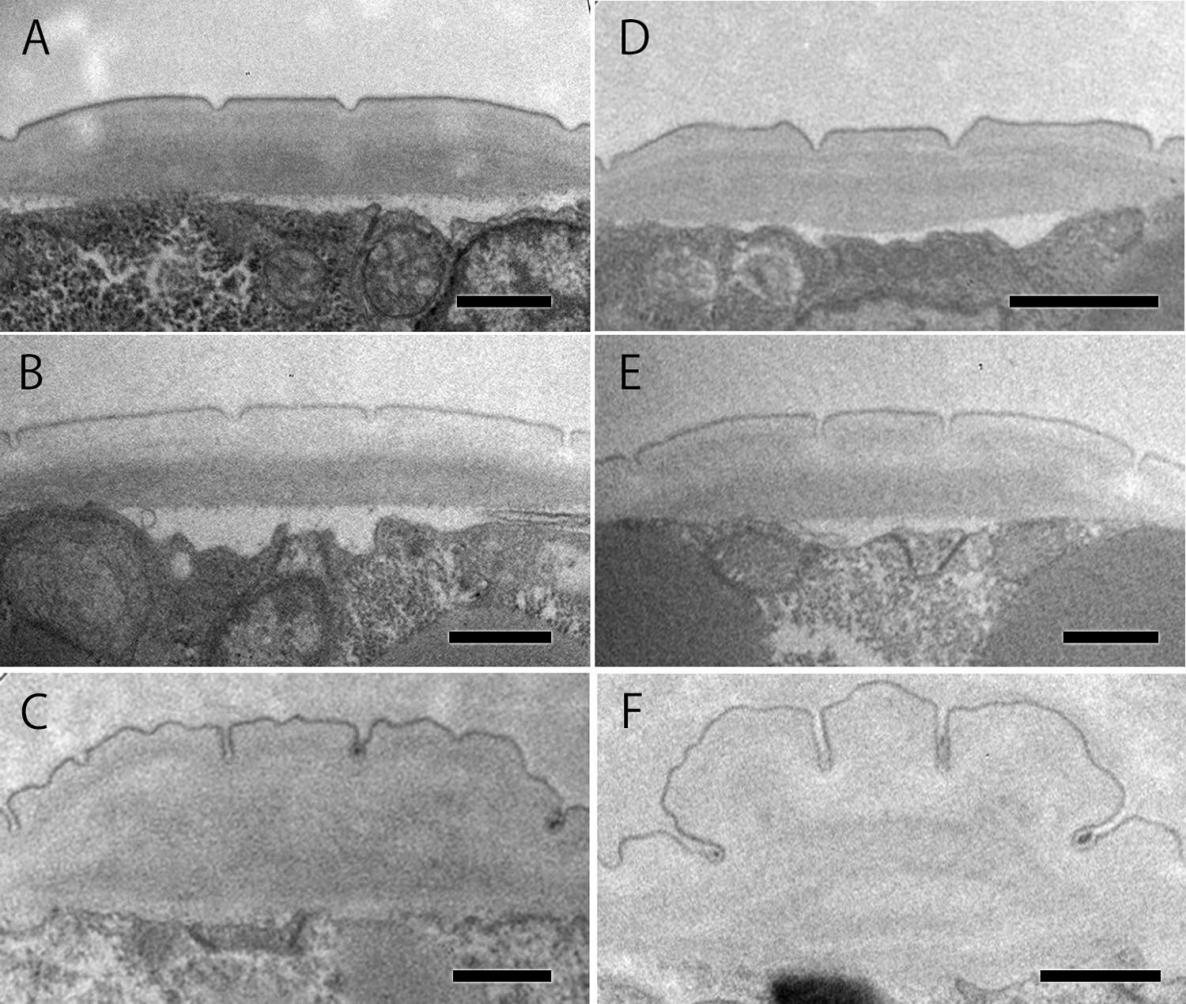
Structure of the lateral alae in *B. xylophilus* adults. (**A**) Female in mycetophagous phase cultured on agar; (**B**) female in mycetophagous phase cultured on pine stem; (**C**) female in phytophagous phase; (**D**) male in mycetophagous phase cultured on agar; (**E**) male in mycetophagous phase cultured on pine stem; (**F**) male in phytophagous phase. Scale bar = 500 nm.

Observing the cuticle structure, we found that the structures of the lateral alae differed markedly between the mycetophagous and phytophagous phases in males. The lateral alae were not conspicuous, i.e., were relatively flattened with a smooth surface, in the mycetophagous phase (Fig. 2D, E) in males and in both phases in females (Fig. 2A–C). On the other hand, the lateral alae were very well developed, with each band expanded with a mushroom-like outline in cross section in the phytophagous phase (Fig. 2F). To evaluate the degree of protrusion from cuticle, the protruding area of lateral alae was measured (Table 1). There were significant differences in the protrusion area between the phytophagous and mycetophagous phases in males cultured on agar plates (P < 0.05) and on pine stems (P < 0.01), whereas no qualitative or quantitative differences were observed in the lateral alae of females between phases.

**Table 1:** Morphological characteristics associated with the phytophagous and mycetophagous phases of *Bursaphelenchus xylophilus.*

Phase	Sex	n	Cuticle Thickness (µm)	Lateral alae Area (µm^2^)
Mycetophagous cultured on agar plates	M	6	0.32 ± 0.03*	0.19 ± 0.21*
Mycetophagous cultured on pine stems	M	10	0.27 ± 0.01	0.18 ± 0.15**
Phytophagous	M	7	0.26 ± 0.04	0.56 ± 0.25
Mycetophagous cultured on agar plates	F	7	0.29 ± 0.03*	0.01 ± 0.24
Mycetophagous cultured on pine stems	F	10	0.26 ± 0.02	0.04 ± 0.11
Phytophagous	F	6	0.26 ± 0.03	0.13 ± 0.32

All numerical values are reported as the means ± standard deviation (SD). The measurements were carried out using ImageJ software version 1.52v (https://imagej.nih.gov/ij/)^41^. *M* male, *F* female, *n* number of replicates. Welch’s t-test was applied to compare the thickness of the cuticle and the area of the lateral alae between the phytophagous and mycetophagous phases (cultured on media plates and pine stems). The significance level was adjusted by the Bonferroni method. Asterisks indicate significant differences between phases (*P < 0.05, **P < 0.01).

### Intestines

**Figure 3:**
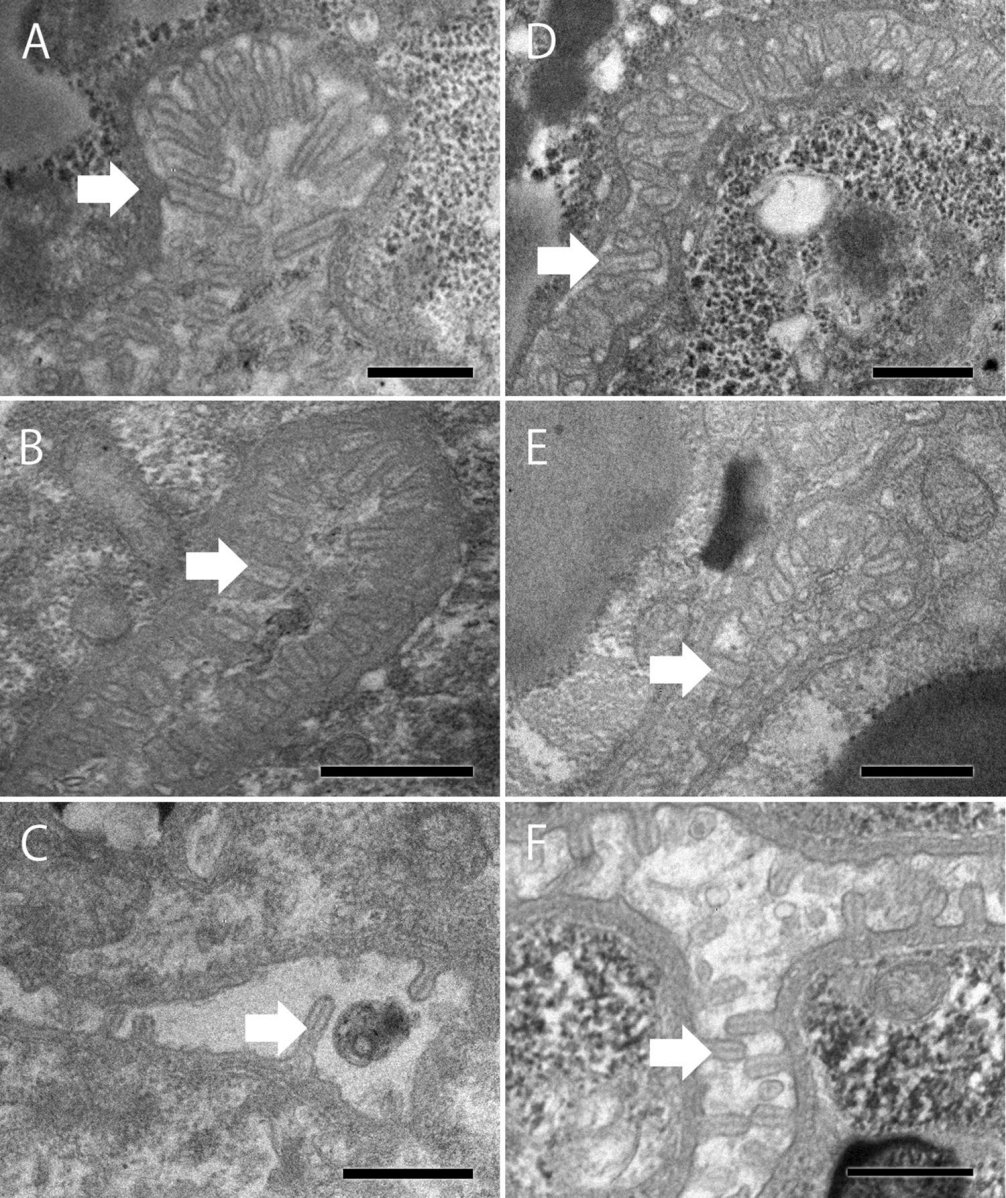
Intestinal structure in *B. xylophilus* adults. (**A**) Female in mycetophagous phase cultured on agar; (**B**) female in mycetophagous phase cultured on pine stem; (**C**) female in phytophagous phase; (**D**) male in mycetophagous phase cultured on agar; (**E**) male in mycetophagous phase cultured on pine stem; (**F**) male in phytophagous phase. The white arrow indicates a microvillus. Scale bar = 500 nm.

All individuals had some microvilli on the internal surface of the intestines (Fig. 3). The microvilli were connected to the basement membrane and supported by an axial core of actin filaments. However, morphological and quantitative characteristics differed between the mycetophagous and phytophagous phases. Microvilli on the internal surface of the intestines were longer and more numerous in the mycetophagous phase (cultured on either agar plates or pine stems) than in the phytophagous phase (Fig. 3).

**Figure 4:**
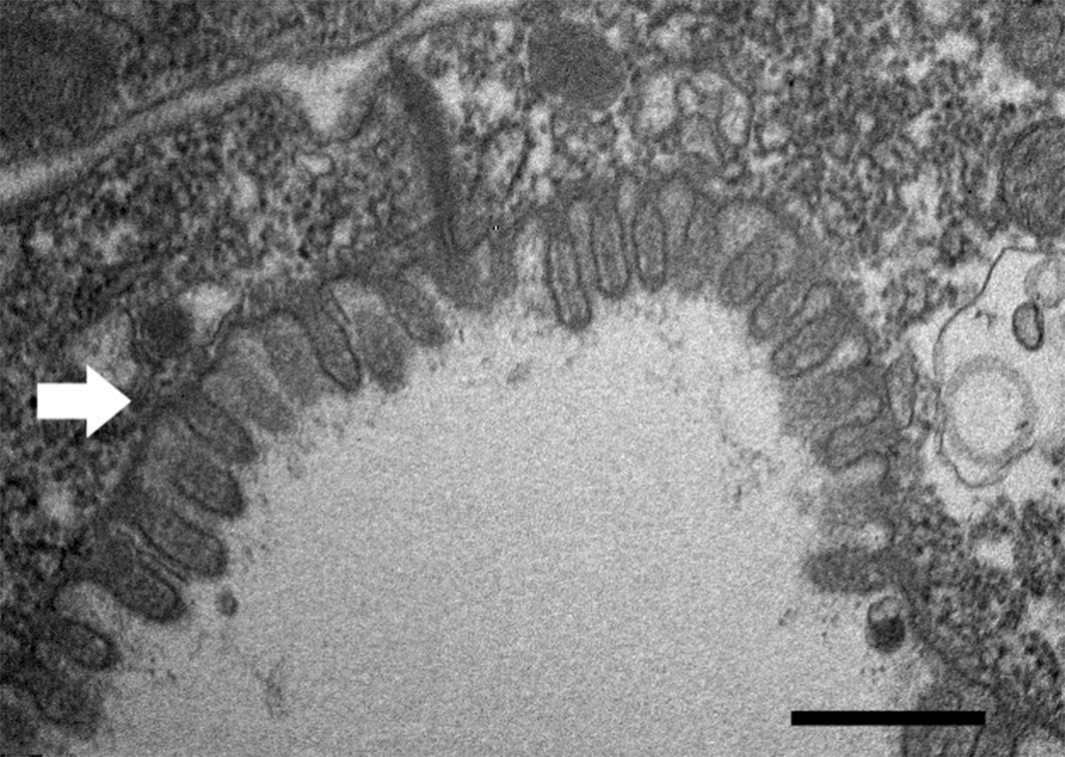
Intestinal structure in *Schistonchus* sp. (undescribed species) female. The white arrow indicates a microvillus. Scale bar = 500 nm.

The intestinal structure of *Schistonchus* sp., an obligate plant parasite, was also observed in this study for comparisons with the structure of the facultative plant parasite *B. xylophilus.* The intestinal structure of *Schistonchus* sp. was similar to that of *B. xylophilus* in the mycetophagous phase; i.e., the microvilli were long and numerous (Fig. 4).

## Discussion

We studied the phenotypic plasticity of *B. xylophilus* to investigate its adaptation strategy in response to the changing environment in pine trees. We focused on its ultrastructure and made comparisons between the mycetophagous and phytophagous phases using TEM. As a result, we revealed that nematodes in the phytophagous phase had more developed lateral alae and more atrophic intestinal microvilli than those in the mycetophagous phase.

**Figure 5:**
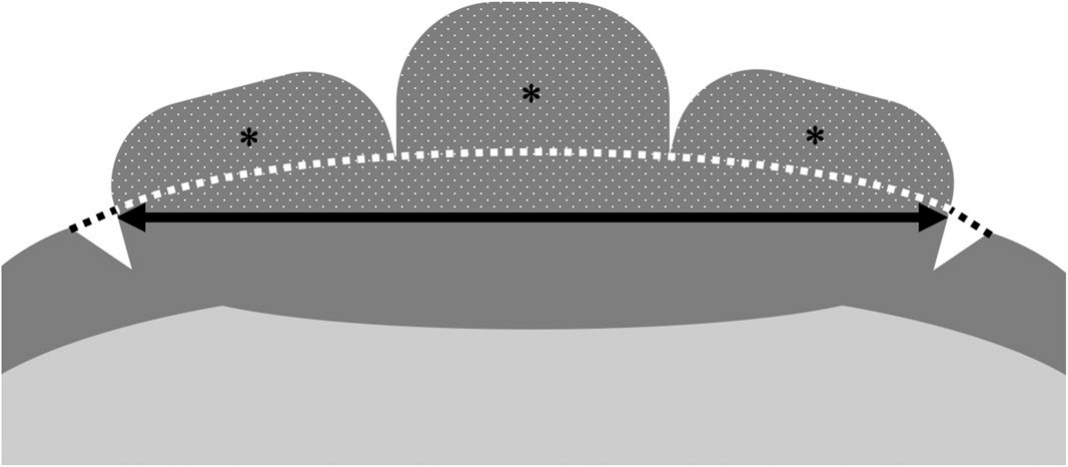
Diagram of the lateral alae illustrating calculation of the degree of protrusion. The area of the lateral alae, indicated by dots, was measured with a polygon-section tool in ImageJ software version 1.52v (https://imagej.nih.gov/ij/)^41^. The width of the lateral alae, indicated by the black arrow, was measured with the straight tool in ImageJ software. The area indicated by asterisks was calculated from these two values.

The lateral alae of nematodes in the phytophagous phase were mushroom-shaped, whereas those of nematodes in the mycetophagous phase were smooth. Shinya et al*.*^26^ observed the surface structure in *B. xylophilus* in the mycetophagous and phytophagous phases using scanning electron microscopy. Although they did not refer to the various forms of the lateral alae, Fig. 5 in their paper showed that nematodes in the phytophagous phase had well-developed lateral alae compared to nematodes raised on fungus. Alae are thickenings or projections of the cuticle that occur in the lateral or sublateral region. Lateral alae occur in both sexes, two per individual, and run longitudinally along the length of the nematode body^27^. They enable the nematode to change shape and the cuticle to flex during dorsoventral contractions. The exact function of the lateral alae remains unknown; however, their structure varies considerably, not only among different taxa but also across developmental stages within a species^27^. Therefore, we hypothesize that the lateral alae are one of the most functionally important structures of the nematode surface features, formed by elaborations of the cuticle^27^. The alae have a complex structure, which differs from that of the general body cuticle, and they may provide a degree of longitudinal stiffening. Furthermore, because nematodes lie and move on their sides^28^, the alae are in contact with the substrate, rather like the tread of a car tire^27^, where they probably assist in locomotion by increasing traction and preventing slipping, although their absence in some forms does not appear to inhibit movement. For example, the lateral alae in the dauer stage in *Caenorhabditis elegans* are different from that in other developmental stages and are mushroom-shaped^29^. Dauer larvae display a specific behavior known as “nictation,” in which the nematodes lift and wave the anterior part of their bodies^13^, enabling them to attach to vectors such as insects^30^. The lateral alae are considered to play an important role in this specific behavior. The alae run longitudinally along the length of the nematode body and are also thought to support the body. It is likely that the alae enable nematodes to undertake three-dimensional activities, such as nictation as well as crawling.

Phytophagous individuals of *B. xylophilus* have well-developed and complex lateral alae. This suggests that *B. xylophilus* moves more actively in a living pine tree than on a dead pine tree and fungal mat. Nematodes were collected from fungal culture plates or pine trees, and phytophagous individuals exhibited three-dimensional activity in water, whereas mycetophagous individuals did not. From a pathological viewpoint, migration ability in a living tree is an indispensable trait for *B. xylophilus* to cause pine wilt disease^31^. *Bursaphelenchus xylophilus* nematodes migrate to the xylem tissues, spreading throughout the infected pine tree, and parenchymatous cell death and cavitation occur following the nematode migration. Finally, the infected pine tree dies due to a lack of water. Ichihara et al*.*^31^ compared the migration between virulent and avirulent strains of *B. xylophilus* in the tissues of living pine trees. They reported that the avirulent strain barely invaded the xylem resin canals and cortical tissue, whereas the virulent strain did. They concluded that the migration of *B. xylophilus* is an important factor that increases the severity of disease symptoms in infected pine trees. Considering these results, we hypothesize that well-developed lateral alae are necessary for *B. xylophilus* to actively move through tree tissues and cause disease.

The intestinal structure of *B. xylophilus* differed between the mycetophagous and the phytophagous phases. Although the number of microvilli could not be counted for technical reasons, the properties of the microvilli were observed. Both females and males had more developed microvilli in the mycetophagous phase than in the phytophagous phase. The microvilli in the mycetophagous nematodes were thicker and longer than those in the phytophagous nematodes. Generally, animals absorb nutrients through their intestines from their food. Previous reports showed that the intestinal and microvilli structure on the internal surface of the intestine changes according to nutritional conditions in many animal species, e.g., chicks^32^, pigs^33^, and pythons^34^. In terms of nematodes, the intestinal lumen in the dauer larvae of *Caenorhabditis elegans* and *B. xylophilus* was small, and the brush border was so compact that individual microvilli were difficult to discern^25,35^.

In the phytophagous phase, *B. xylophilus* most likely uses epithelial cells and the resin canal parenchyma cells as food sources in living trees. Given that the intestinal microvilli of *B. xylophilus* are shrunken in the phytophagous phase, epithelial cells of the living pine tree are not considered suitable food for *B. xylophilus*. This coincides with the fact that the reproductive rate of *B. xylophilus* on fungal mats (e.g., *Botrytis cinerea*) is higher than in pine seedlings^19,36^.

To understand the intestinal adaptation of *B. xylophilus* to the host plant in a phylogenetic context, we observed the intestinal ultrastructure of an undescribed *Schistonchus* sp. This species is an obligate plant-parasitic species and closely related to *B. xylophilus*, i.e., both species belong to the family Aphelenchoididae^24^. Interestingly, *Schistonchus* sp. feeding on plant (fig) tissues had well-developed microvilli on the surface of the intestine. It was also reported that the L2 and L3 of the obligate plant-parasitic nematode *Heterodera glycines* had a moderate proliferation of microvilli^37^. These results suggest that plant-parasitic species, including *Schistonchus* sp., can ingest sufficient nutrients from plant tissues. *Schistonchus* share an ancestor with *B. xylophilus* and is physiologically adapted as a plant parasite, indicating that nematodes are genetically plastic. Considering that the ancestral species of *B. xylophilus* is a mycetophagous species, it appears that *B. xylophilus* is in the process of adapting to being able to feed on plant cells.

In this study, we showed that *B. xylophilus* was able to alter its ultrastructure according to the environment. This is the first report of phenotypic plasticity in *B. xylophilus* at the ultrastructural level. The lateral alae were more developed in the phytophagous phase than in the mycetophagous phase. On the other hand, the intestine was less developed in the phytophagous phase than in the mycetophagous phase. The ultrastructure in the phytophagous phase was similar to that at the dauer stage, enabling the nematode to survive harsh environments. This suggests that ultrastructural phenotypic plasticity in *B. xylophilus* is a strategy for surviving harsh environments such as those encountered while actively migrating within the living pine tree instead of feeding. This rapid dispersion, regulated by the phenotypic plasticity, could cause the rapid development of symptoms observed in infected pine trees. To date, little research has focused on phenotypic plasticity in *B. xylophilus*^20,38^. However, investigating phenotypic plasticity is essential to aid our understanding of the survival strategy of *B. xylophilus*, and eventually, the survival strategy of Nematoda.

## Materials and methods

### Nematode preparation

Mycetophagous and phytophagous phases were prepared. To understand the effects of growth medium, the mycetophagous phase was cultured on two grown media, i.e., agar plates and dead pine stems.

### Mycetophagous phase cultured on agar plates

The *B. xylophilus* Ka4 C1 strain was cultured with *Botrytis cinerea* on malt extract agar plates (1.5% malt extract and 4.0% agar) at 25 °C. Nematodes were collected using the Baermann funnel technique overnight. Nematodes were rinsed three times with ion-exchange water (IEW), and females and males were randomly harvested.

### Mycetophagous phase cultured on pine stems

Mycetophagous phase growing on pine twigs was prepared following Kanzaki et al.^17^. The *B. xylophilus* Ka4 C1 strain was cultured as described above. Ten stems (ca 7 mm in diam. and 4 cm long) of 3-year-old Japanese black pine (*Pinus thunbergii*) were autoclaved and individually transferred to 15-mL sterile plastic centrifuge tubes. *B. cinerea* was inoculated onto the stems. One week after inoculation, 500 nematodes were inoculated into the tubes. At 2 weeks after inoculation, the nematodes were extracted from the wood using the Baermann funnel technique overnight. The extracted nematodes were lumped together, transferred to a 15 mL sterile plastic centrifuge tube, and rinsed three times with IEW. Then, females and males were randomly harvested.

### Phytophagous phase

The *B. xylophilus* Ka4 C1 strain was cultured as described above. Nematodes were washed three times with IEW and adjusted to 10,000 individuals per 500 µL of IEW. Two-year-old Japanese black pine trees were each inoculated with 10,000 nematodes on April 25, 2019. Four pine trees were inoculated in all. At 2, 3, and 4 weeks after inoculation, nematodes were extracted from two, one, and one pine tree, respectively, using the Baermann funnel technique overnight. The extracted nematodes were lumped together, and transferred to a separate 15-mL sterile plastic centrifuge tube for each week, rinsed three times with IEW, and females and males were randomly harvested.

### Obligate plant-parasitic species closely-related to *Bursaphelenchus xylophilus*

Fruits of *Ficus superba* were collected in June 2017 from Ishigaki Island, Okinawa, Japan. A collection permit was not necessary for *F. superba* because it was not obtained inside a protected area. The fig fruits were identified visually and dissected under a light microscope (S8 Apo; Leica). Parasitic females of an undescribed *Schistonchus* sp. were identified under the light microscope (Eclipse 80i; Nikon; 200× or 400×) and used for the ultrastructural observations.

### Observation of ultrastructure using TEM

Samples were prepared for TEM as described by Ekino et al.^39^ Adult nematodes were fixed in 1.25% glutaraldehyde and 1.5% picric acid (only 1% glutaraldehyde was used for fixing *Schistonchus* sp.) in 0.1 mol/L phosphate buffer (pH 7.4) for more than 24 h. Then, the heads and tails were excised from the fixed adults, and the mid-body regions were used; almost all positions were of equal thickness. The nematodes were arranged in a parallel array on a 2% agar pad prepared on a microscope slide^40^. Molten 2% agarose was dripped onto the pad containing three to five nematodes. After the agarose had solidified, we trimmed it into a cube and dripped molten 2% agarose to cover the surfaces of the agarose cube. It was then trimmed again to form a larger cube. Several cubes were prepared for each nematode species. The cubes were rinsed six times with phosphate buffer (10 min for each rinse) and post-fixed in 1% osmium tetroxide in IEW for 90 min. Then, the samples were dehydrated in a graded series of ethanol baths (one bath each of 50%, 70%, 80%, and 90% ethanol, and three baths of 99.5% ethanol), and cleaned three times with propylene oxide for 5 min. The samples were infiltrated overnight with a mixture of 50% Eponate resin and 50% propylene oxide. On the following night, they were infiltrated with undiluted resin. Finally, the samples were embedded in Epon resin.

We used a diamond knife fitted to an ultramicrotome to section the mid-body region of the nematodes. Sections were only used when the nematodes were cut vertically along the long axis of the body. The sections were collected on Formvar-coated copper grids for electron microscopy. The grid was stained with EM Stainer (Nissin EM, Tokyo, Japan) for 30 min, followed by lead citrate. Grid-mounted sections were photographed at 100 kV using a JEOL JEM-2010 electron microscope (Tokyo, Japan). One section in which the structures were observed clearly in each nematode was used for measurement. The thickness of the total cuticle and the total cross section area were measured from these cross-sections using ImageJ Software version 1.52v (National Institutes of Health, Bethesda, Maryland, USA)^41^. The cuticle was measured once where the cuticle structure was observed clearly and outside the annuli. The body radius (*r*) was calculated from the total cross-section area.

To evaluate the degree of protrusion of lateral alae from the cuticle, the protrusion area (*S*; areas indicated by asterisks in Fig. 5) was calculated. First, the area of the lateral alae (*S′*; area indicated by dots in Fig. 5) was measured with a polygon-section tool in ImageJ, and their width of (*x*; arrow in Fig. 5) was measured with the straight-tool in ImageJ. *S* was calculated using the following formula:

$$ S = S^{\prime} - \left( {\theta x^{2} - \frac{xr\cos \theta }{2}} \right), \theta = \sin^{ - 1} \frac{x}{2r}. $$

### Statistical analysis

Significant differences in cuticle thickness and protrusion area of lateral alae between the phytophagous and mycetophagous phases (cultured on media plates and on pine stems) were identified using Welch’s t-test. The significance level was adjusted uisng the Bonferroni method. The procedure was performed using Excel 2013 for Windows (Microsoft Corporation, Redmond, WA, USA). A value of P < 0.05 was considered to indicate statistical significance.
